# Covalent organic polymer induces apoptosis of liver cancer cells *via* photodynamic and photothermal effects

**DOI:** 10.3389/fonc.2022.986839

**Published:** 2022-11-09

**Authors:** Wenze Xu, Mengfan Zhang, Wenhui Wang, Manzhou Wang, Bingjie Li, Hao Li, Donglin Kuang, Chao Liang, Jianzhuang Ren, Xuhua Duan

**Affiliations:** ^1^ Department of Interventional Radiology, First Affiliated Hospital of Zhengzhou University, Zhengzhou, Henan, China; ^2^ Department of Oncology, First Affiliated Hospital of Zhengzhou University, Zhengzhou, Henan, China

**Keywords:** photodynamic therapy, photothermal therapy, supramolecular materials, hepatocellular carcinoma, tumor therapy

## Abstract

The purpose of this study was to explore the photodynamic and photothermal effects of the supramolecular material Purp@COP and to test the anti-cancer effect on HepG2 cells *in vitro*.

**Materials and methods:**

Purp@COP is a covalent organic polymer (COP) with robust tailoring heteroatom incorporation, plentiful pore structure, and multiple functions similar to the metal–organic framework (MOF). Hepatocellular carcinoma cell line HepG2 was cultured with Purp@COP for 24 h and treated with near-infrared 808-nm laser 1 W/cm^2^ for 10 min. Cell Counting Kit-8 (CCK-8) assay, colony formation assay, live–dead cell fluorescence staining, and Annexin V/propidium iodide (PI) staining flow cytometry were performed to detect the viability, proliferation, and apoptosis of the HepG2 cells.

**Results:**

The supramolecular material Purp@COP exhibited significant photothermal performance under near-infrared 808-nm laser irradiation *in vitro*. With the treatment of Purp@COP and near-infrared 808-nm laser irradiation on HepG2 cells, cell viability and colony formation capacity were decreased, and the number and proportion of apoptotic cells were increased.

**Conclusions:**

The supramolecular material Purp@COP has both photothermal and photodynamic effects and can significantly induce cancer cell death and inhibit the proliferation of cancer cells *in vitro*.

## Introduction

Nowadays, because of its high thermal stability and precisely controllable capacities, the covalent organic polymer attracts tremendous interest as a class of ever-growing porous materials. The rich nitrogen coordination sites in covalent organic polymer (COP) materials could stabilize single-atom metals and provide efficient mass transport (1). Currently, there are two main mechanisms of phototherapy: photodynamic therapy (PDT) and photothermal therapy (PTT). The former exerts local chemical damage in the target area, and the latter induces local thermal damage. PDT is a phenomenon in which in a photosensitive material (photosensitizer (PS)) with high affinity for tumor tissues under laser radiation, the PS absorbs energy and transmits energy to the surrounding oxygen molecules. The reactive oxygen species (ROS) and free radicals are generated in this way and interact with the cellular membrane, destroying the structure and distorting the function of cellular organelles, consequently leading to cell death of cancers (2, 3). In clinical practice, photodynamic therapy has been approved in various countries for some superficial tumors, including skin tumors (4), superficial bladder tumors (5), lung tumor orifices (6), gynecological tumors (7), and head and neck tumors (8). PTT is a phenomenon in which materials absorb light energy and convert it into heat energy, which induces a lethal effect on cancer cells by hyperthermia. In this process, the absorbed light is transferred through electron–electron and electron–phonon relaxation and then forms a thermal nanoparticle (NP) lattice. The NP structure is cooled down through phonon–phonon relaxation, and enhanced thermal scattering leads to an increase in the temperature of cancer cells near NPs (9). The hyperthermia in turn causes cell lysis and enzyme release, leading to protein denaturation and cell death.

PS is one of the core elements of PDT. The first generation of PSs is represented by porphyrins, including their derivatives hematoporphyrin (HpD) and porphine. They have disadvantages such as poor affinity for tumor tissues, low absorption of near-infrared light, and long elimination half-life time. The second-generation PSs represented by protoporphyrin and phthalocyanine overcome some inherent defects of the first-generation PSs and acquire enhanced tumor targeting ability, can be excited at a longer wavelength, and have shorter elimination half-life and higher efficiency, higher chemical purity, singlet oxygen generation rate, and deeper tissue penetration (10). However, the disadvantages of the second-generation PSs including low water solubility, low targeting efficiency, and poor tumor degeneration remain to be further improved (10–12). Studies have found that many nanomaterials can produce ROS with photo-excitation, which can be used as novel nano-PS (13, 14). Compared with traditional organic small-molecule PS, nano-PS has excellent photostability and photosensitive activities and accessibility. A study has shown that ultrathin black phosphorus nanosheets with a thickness of 2.0 nm and Cu^2+^-modified carbon nitride nanosheets (Cu^2+^-g-C_3_N_4_) as a nano-PS had a potent anti-cancer effect (15).

In addition to PSs, the light source is another core element of PDT. Most excitation wavelengths of PS are in the UV or visible spectrum, but these light sources have shallow tissue penetration depth and cannot meet the requirements of deep tumor treatment, which limits the therapeutic effect of PDT. Compared with UV light or visible light, near-infrared light (700–1,300 nm) has a deeper tissue penetration capacity. Depending on different human tissues, the penetration depth of near-infrared light is up to 10 mm, which is more appropriate for PDT in the treatment of deep solid tumors *in vivo* (16).

In view of the fact that the sole effect of PTT or PDT is often unsatisfactory, the creation of PSs, which have both effects, has been proposed. However, there are few studies on supramolecular materials with dual properties. Nanocrystalline supramolecular materials have been obtained using the developed edge confined route according to previous work (17, 18) on Purp@COP, which have both photodynamic and photothermal effects. The material includes both the main body and the object parts. The main body COP is the basic framework, and the object Purp is methyl viologen (1,1′-dimethyl-4,4-bipyridinium dichloride (6Cl,7Cl)), which is anchored to the COP by the physical load. This study aimed to investigate the photodynamic and photothermal effects and the antitumor effects of the synthesized supramolecular material Purp@COP using *in vitro* assays on HepG2 cells.

## Experimental

### Material synthesis and detection

COP was prepared according to previous work (17, 18). During the synthesis, Pt^2+^ restricts the spatial position of the ligand through coordination interactions, forming a fused quasi-phthalocyanine backbone with a platinum coordination center with the obtained covalent organic framework. Purpurine was then mixed at room temperature for 3 h to obtain Purp@COP. Relevant characterization of this material was performed by transmission electron microscope (TEM) (model Titan G260-300) and dynamic light scattering particle size analyzers (Malvern Zetasizer Nano ZS90, UK).

### Photothermal effect

Dissolve Purp@COP in water at the concentration gradient of 0, 50, 100, 200, and 400 μg/ml; take the same volume and irradiate at 1 W/cm^2^ under the near-infrared 808-nm laser (MDL-XF-808 nm-10W-BJ00313) for 10 min; and determine the temperature rise through the laser’s own infrared thermometer.

### Cell culture and identification

HepG2 cells were purchased from the China Center for Type Culture Collection and cultured with Dulbecco’s modified Eagle’s medium (DMEM) culture medium (Gibco, Thermo Fisher Scientific, Waltham, MA, USA) containing 10% fetal bovine serum (Gibco, Thermo Fisher Scientific), 100 U/ml of penicillin, and 10 µg/ml of streptomycin in a 37°C incubator with 5% CO_2_.

### Cell Counting Kit-8 assay

The tumor cell line HepG2 was uniformly seeded on a 96-well plate with an attachment time of 24 h. Cells were cultured with new DMEM and treated with Purp@COP at the following concentrations: 0, 10, 20, 50, and 100 μg/ml. After 24 h of incubation in the incubator, 10 μl of Cell Counting Kit-8 (CCK-8) solution (Vazyme, Nanjing, China) was added to each well and incubated for 1.5 h at 37°C. The 96-well plate was measured at 450 nm according to a microplate reader (spectraMax-i3). The optical density of each well was normalized and calculated.

### Colony formation assay

HepG2 were uniformly seeded on six-well plates at a density of 1,000 cells per well for an attachment time of 24 h. Cells were cultured with new DMEM and treated according to the following groups: DMEM group, Purp@COP alone (50 μg/ml) group, laser alone (1 W/cm^2^; 10 min) group, and Purp@COP + laser group. They were incubated in a 37°C incubator, and after 72 h, crystal violet staining was performed, and photos were taken for observation.

### Live–dead cell staining

Live–dead cell staining was performed according to the operating procedures of the live–dead cell staining kit (BioScience, Shanghai, China). After cell culture, cells were treated according to the following groups: DMEM group, Purp@COP alone (50 μg/ml) group, laser alone (1 W/cm^2^; 10 min) group, and Purp@COP + laser group. At the end of the treatment, the supernatant was removed and incubated with a new serum-free DMEM culture medium containing 2 μM of calcein acetoxymethyl ester (calcein AM) and 4.5 μM of propidium iodide (PI) for an additional 30 min. The cells were observed under a fluorescence microscope (Leica DFC450, Wetzlar, Germany).

### Reactive oxygen species staining

ROS staining was performed according to the operating procedures of the DHE kit (BioScience, China). After cell culture, cells were treated according to the following groups: DMEM group, Purp@COP alone (50 μg/ml) group, laser alone (1 W/cm^2^; 10 min) group, and Purp@COP + laser group. At the end of the treatment, the supernatant was removed, and incubation was continued for 30 min with a new serum-free DMEM culture medium containing 5 μM of dihydroethidium (DHE). The cells were observed under a fluorescence microscope (Leica DFC450).

### Flow cytometry

Flow cytometry was performed according to the operating procedures of FITC-Annexin V and PI Apoptosis kit (BioScience, China). After cell culture, cells were treated according to the following groups: DMEM group, Purp@COP alone (50 μg/ml) group, laser alone (1 W/cm^2^; 10 min) group, and Purp@COP+ laser group. At the end of the treatment, cells were collected and incubated with the working solutions containing FITC-Annexin V and PI for 15 min at room temperature in the dark. Analysis was performed by cell analyzer (BD FACSCelesta™, San Jose, CA, USA).

### Western blotting analysis

Protein samples were prepared in lysis buffer (HEPES 25 mmol/L, KAc 150 mmol/L, EDTA pH 8.0 2 mmol/L, NP-40 0.1%, NaF 10 mmol/L, phenylmethylsulfonyl fluoride (PMSF) 50 mmol/L, aprotinin 1 µg/µl, pepstatin 1 µg/µl, leupeptin 1 µg/µl, and DTT 1 mmol/L). Protein concentration was quantified by bicinchoninic acid (BCA) protein assay (Beyotime, Shanghai, China) according to the protocol using bovine serum albumin (BSA) to prepare a standard curve. Gel electrophoresis was performed with 10–20 µg of protein using 4%–15% gels (Beyotime, China), followed by transblotting to 0.2-µm nitrocellulose membrane (Amersham, Piscataway, NJ, USA). Protein band intensities were determined and detected with BeyoECL Star (Beyotime, China) using the Amersham Imager 680 system (GE). Primary antibodies used in the experiments, including anti-Bcl-2 (rabbit mAb, Cell Signaling, Danvers, MA, USA) and β-actin (rabbit mAb, Cell Signaling, USA) were diluted 1:1,000 in 1% BSA. The secondary antibody used in the experiments including horseradish peroxidase (HRP)-conjugated goat anti-rabbit IgG (Beyotime, China) was diluted at 1:1,000.

## Results

### Purp@COP characterization

**Figure 1:**
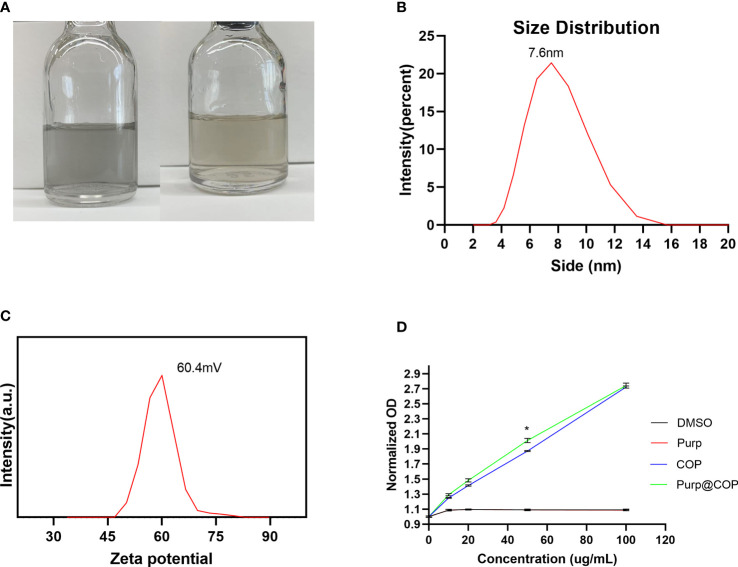
Solubility test. **(A)** The photo of Purp@COP dispersed in water for 1 day (left) and 2 weeks (right). **(B)** Size distribution of Purp@COP in water at 25°C. **(C)** Zeta potential of Purp@COP in water at 25°C. **(D)** The absorption intensity at 808 nm for Purp, COP, and Purp@COP. The p value is less than 0.05, meaning that there is statistically significant.

Purp@COP demonstrated good solubility in aqueous solution and could form a stable aqueous solution for more than 2 weeks (**Figure 1A**). The as-observed results displayed Purp@COP with an average diameter of 7.6 nm (**Figure 1B**). Purp@COP demonstrated a stable zeta potential (**Figure 1C**). The results of absorption intensity at 808 nm for Purp, COP, and Purp@COP showed that Purp@COP has a good light absorption ability (**Figure 1D**).

**Figure 2:**
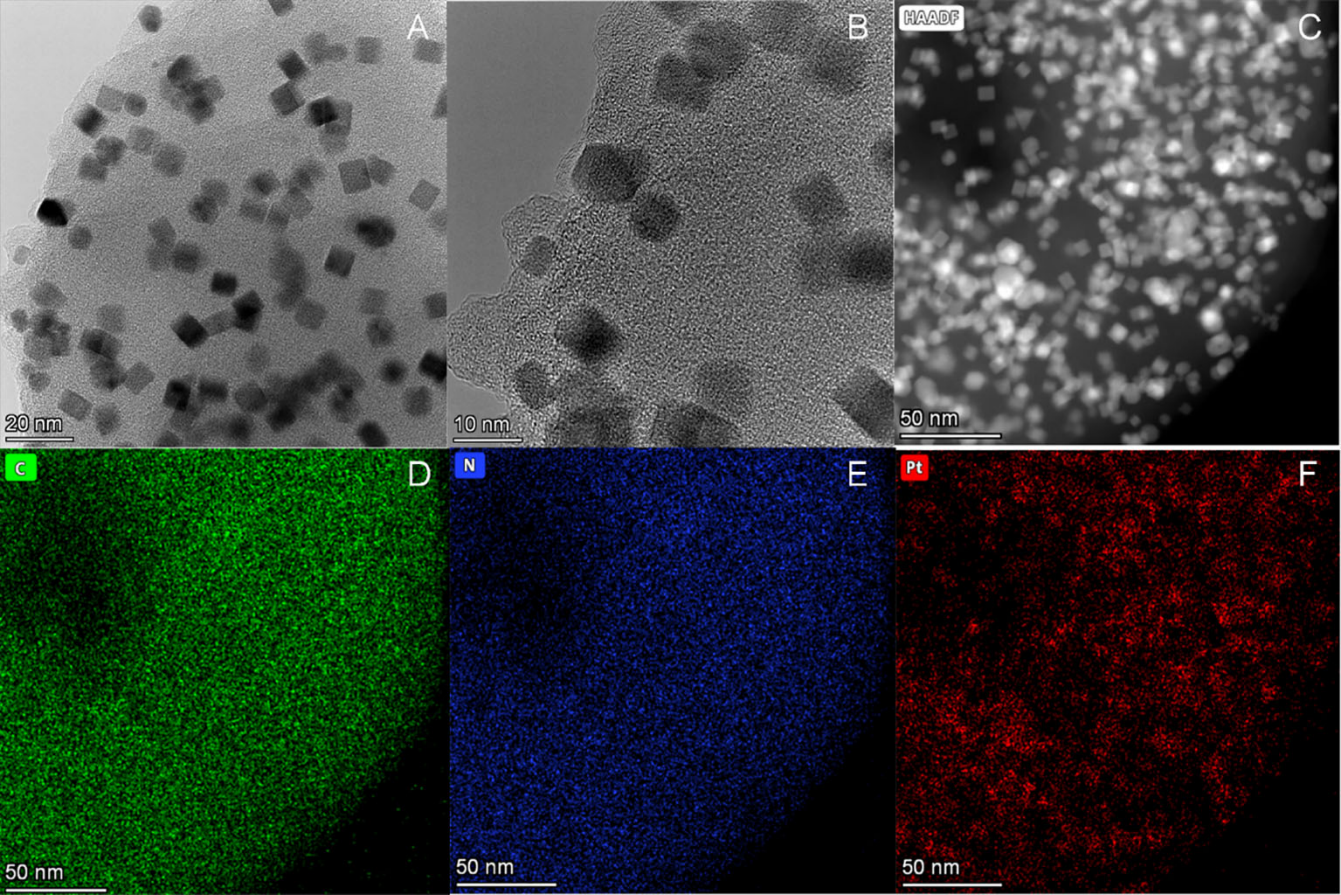
Structural and morphological characterization of Purp@COP. **(A)** Transmission electron microscope (TEM) images of Purp@COP (scale, 20 nm). **(B)** High-resolution TEM images of Purp@COP (scale, 10 nm). **(C)** High-angle annular dark-field scanning transmission electron microscope (HAADF-STEM) of Purp@COP (scale, 50 nm). **(D–F)** Energy-dispersive spectrum (EDS) elemental mapping images of C, N, and Pt (scale, 50 nm).

Under the TEM, it was clearly observed that the nano-platinum was uniformly distributed (**Figure 2A**). The high-resolution electron microscope clearly showed the edge of the material, with all the nano-platinum inside (**Figure 2B**). Further results observed with a high-angle annular dark-field scanning transmission electron microscope (HAADF-STEM) showed that the dispersed platinum nanoclusters were evenly distributed throughout the surface (**Figure 2C**). The associated energy-dispersive spectrum (EDS) elemental maps further confirmed that the Pt nanoclusters were highly distributed along the backbone (**Figures 2D–F**).

### Purp@COP has a good heating effect

**Figure 3:**
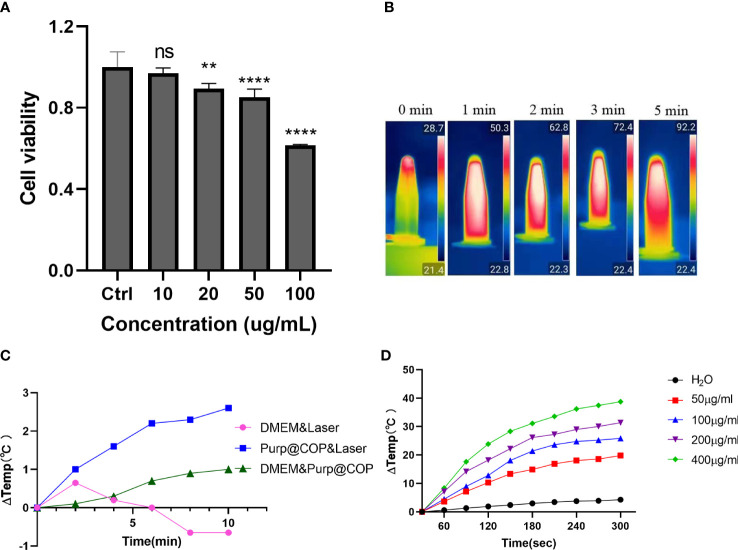
Heating effect results of Purp@COP. **(A)** Cell activity results of HepG2 cells after culture for 24 h with 10, 20, 50, and 100 μg/ml of Purp@COP. **(B)** Heating curve after irradiation with 1 W/cm^2^ laser with or without 50 μg/ml of Purp@COP in a common culture dish. **(C)** Heating results of Purp@COP under laser (1 W/cm^2^) irradiation. **(D)** The heating results of different concentrations of Purp@COP under laser (1 W/cm^2^) irradiation. The symbols "**", "****" showed that the p value is less than 0.05,while "ns" showed p>0.05.

HepG2 cells were treated with Purp@COP at concentrations of 0, 10, 20, 50, and 100 μg/ml, and there was no significant cytotoxic effect when the concentration was lower than 50 μg/ml (**Figure 3A**). The heating effect of Purp@COP dissolved in the medium under laser radiation or not (**Figure 3B**). With laser radiation, Purp@COP could be heated to 92.2°C within 5 min (**Figure 3C**). The concentration gradient results of Purp@COP showed that its heating curve was positively correlated with the material concentration (**Figure 3D**).

### The photodynamic effect of Purp@COP can effectively promote apoptosis and necrosis and significantly inhibit the proliferation ability of HepG2 cells

**Figure 4:**
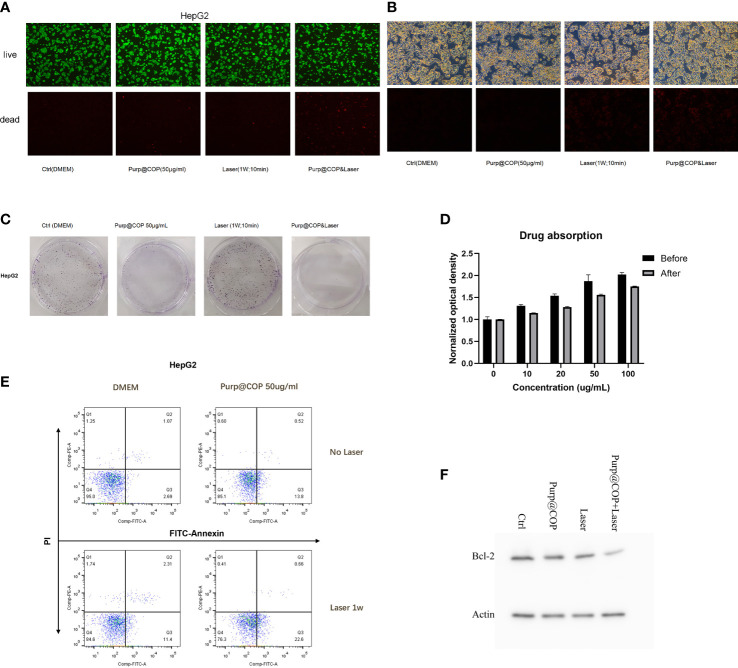
Antitumor results of the photodynamic and photothermal effects of Purp@COP. **(A)** HepG2 cells were treated with or without 50 μg/ml of Purp@COP followed by irradiation with or without 1 W/cm^2^ laser for 10 min and then cultured for 24 h before calcein AM and propidium iodide (PI) staining (scale, 20 μm). **(B)** Results of dihydroethidium (DHE) staining after HepG2 cells were treated with or without 50 μg/ml of Purp@COP followed by irradiation with or without 1 W/cm^2^ laser for 10 min (scale, 20 μm). **(C)** Flow cytometry results of Annexin V and PI staining after HepG2 cells were treated with or without 50 μg/ml of Purp@COP followed by irradiation with or without 1 W/cm^2^ laser for 10 min and then cultured for 24 h. **(D)** Results of crystal violet staining after HepG2 cells were treated with or without 50 μg/ml of Purp@COP followed by irradiation with or without 1 W/cm^2^ laser for 10 min and then cultured for 72 h. **(E)** The drug absorption of HepG2 cells before or after the culture with different doses of Purp@COP. **(F)** Protein expression of Bcl-2 of HepG2 cells treated with or without 50 g/ml of Purp@COP followed by irradiation with or without 1 W/cm^2^ laser for 10 min and then cultured for 24 h. Actin was used as a loading control.

HepG2 cells treated with 50 μg/ml of Purp@COP showed a decrease in the number of calcein AM-positive cells and an increase in the number of PI-positive cells after laser radiation, showing that the photodynamic effect of Purp@COP could induce cell death (**Figure 4A**). The results of ROS staining showed that Purp@COP induced massive ROS production in HepG2 cells after laser radiation (**Figure 4B**). The flow cytometry results of FITC/PI double staining showed that Purp@COP combined with laser irradiation induced apoptosis of HepG2 cells, and the proportion of apoptotic cells increased up to 23.26% (**Figure 4C**). To further verify whether Purp@COP combined with laser irradiation had an inhibitory effect on the proliferation of HepG2 cells, a colony formation assay was performed. The results showed that Purp@COP inhibited the proliferation of HepG2 cells, while Purp@COP combined with laser irradiation further enhanced its inhibition on the proliferation of HepG2 cells (**Figure 4D**). The absorbance of culture supernatant after HepG2 cells were cultured for 24 h was compared with that of the supernatant of the same concentrations of 10, 20, 50, and 100 μg/ml before the culture, which showed that the drug could be absorbed well (**Figure 4E**). Protein expression of Bcl-2 was evidently decreased in Purp@COP-treated HepG2 cells after laser radiation, which demonstrated that under laser irradiation, Purp@COP could induce apoptosis of HepG2 cells (**Figure 4F**).

## Discussion

The results show that Purp@COP has satisfactory photothermal and photodynamic effects. In *in vitro* assays, Purp@COP can promote excessive production of ROS in HepG2 cells under laser irradiation, which in turn induces apoptosis and inhibits the proliferative potential of HepG2 cells.

In both phototherapies, PTT exerts a hyperthermia effect in cancer cells through photothermal conversion. Hyperthermia can be used to prompt drug release from heat-sensitive preparations, thereby further improving the accumulation, distribution, and efficacy of chemotherapy (19). However, photothermal agents have unequal photothermal conversion efficiencies, which have become a key factor affecting the therapeutic effect. Most of the clinical research on PTT focuses on the invention of integrated laser device ablation systems. Nowadays, the invention and screening of new photothermal agents have become a hotspot in preclinical research, and then the use of gold nanoshells and related light-absorbing nanomaterials as preclinical models of PTT photothermal agents, etc., has begun to emerge (20, 21). Compared with PTT, PDT has drawn more attention because of its low toxicity and less invasiveness in the treatment procedure of cancers. PDT effect consists of three core elements: PSs, the light at specific wavelengths, and oxygen (22, 23). The combination of different photosensitizers and the application of light sources will increase the complexity of PDT, which affects its therapeutic effects (24). In addition to the above two core elements, similarly, oxygen deficiency can significantly impair the therapeutic effect of PDT (23). In our study, Purp@COP was designed to be synthesized. During the synthesis of the material Purp@COP, Pt^2+^ restricts the spatial position of the ligand through coordination interactions, forming a fused quasi-phthalocyanine backbone with a platinum coordination center. Owing to the light absorption properties of the phthalocyanine backbone, Purp@COP shows excellent photothermal performance (**Figures 3B,C**). Cancer cells are sensitive to heat, and the photothermal properties of Purp@COP warrant its role as a photothermal agent that can be used for the local–regional treatment of cancer. At the same time, Purp@COP has a photodynamic effect and promotes excessive production of ROS (**Figure 4B**), which in turn synergistically eliminates cancer cells with its PTT effect.

Among the practical applications of both phototherapies, photothermal conversion efficiency and oxygen deficiency-induced formation are the main limiting factors (25). The duality of PDT and PTT effects seems to compensate for each other’s drawbacks. Zhang et al. (26) synthesized a multifunctional nano-system with Pd, Au, Ce6, and mesoporous MnO_2_. The synthesized nano-system showed strong a light absorption ability and satisfactory effects of photothermal ablation in solid tumors. AAM HNS_S_ nanoparticles were synthesized by encapsulating Ce6 in Au/Ag-modified hollow mesoporous MnO_2_ absorbed in the near-infrared (NIR) II region by Wu et al. (27). ROS generation catalyzed by MnO_2_ significantly promoted the absorption of the laser by the system, indicating that PDT has a synergistic effect to promote PTT. Sun et al. (28) prepared nanoparticles by encapsulating porphyrin IV in caspase-3-sensitive polymer materials. Under the process of PTT, porphyrin IV releases a large amount of caspase-3 during the photothermal ablation of tumors, which in turn triggers the degradation of polymer materials to release inside porphyrin IV and exerts the PDT effect. Collectively, the duality of PDT and PTT can be achieved in various ways with nanomaterials. In this study, the synthesized Purp@COP was used for biomedical verification. The quick temperature rise effect of Purp@COP with laser radiation (**Figure 3**) demonstrated its satisfactory photothermal properties. The results of live–dead cell assay, ROS staining, flow cytometry of FITC/PI double staining, and colony formation assay (**Figure 4**) showed that the photodynamic effect of Purp@COP could effectively induce cell death and significantly inhibit the proliferation ability of HepG2 cells. The results showed that Purp@COP had both properties, which was equivalent to the combination of both phototherapies.

In clinical treatment, parameters such as the size, morphology, surface modification, power density, and wavelength of near-infrared spectroscopy also have an important impact on the therapeutic effect and efficacy of photothermal and photodynamic therapy techniques (29). The penetration depth of visible light in biological tissues is only 1–2 mm. Compared with the visible wavelength, the fluorophores emitted in the near-infrared region I (NIR-I) can provide relatively deep penetration and higher imaging quality (30). Due to the inherent advantages, such as strong tissue penetration, small tissue absorption and emission light scattering, and low autofluorescence, the near-infrared 808-nm laser was selected for this study, which is effective in minimizing the interference of biological light absorbers considering that *in vivo* fluorescence imaging uses near-infrared light with a longer wavelength (650 – 900 nm) rather than ultraviolet or visible light (31, 32). According to the results, Purp@COP had satisfactory photothermal and photodynamic effects under near-infrared laser irradiation, which indicates high compatibility with the current clinically approved laser generator.

In conclusion, Purp@COP has dual properties of photothermal and photodynamic effects to induce cell death and inhibit the proliferation of HepG2 cells *in vitro*. Therefore, Purp@COP is a very promising photosensitizer for the clinical treatment of solid cancers. However, further studies should be performed to investigate the effect of Purp@COP *in vivo*.

## Data availability statement

The original contributions presented in the study are included in the article/Supplementary Material. Further inquiries can be directed to the corresponding authors.

## Author contributions

XD, JR, BL, and MZ contributed to the conception and design of the study. WX, WW, MW, and CL contributed to the conception of the experiments. BL performed the synthesis and the characterization. MZ, BL, HL, and DK contributed to the analysis of the experimental results. WX, MW, and MZ wrote the first draft of the manuscript. XD and BL wrote sections of the manuscript. All authors contributed to the manuscript revision and read and approved the submitted version.

## Funding

This work was supported by the National Natural Science Foundation of China (No. U2004119), the National Natural Science Foundation of Henan Province (No. 202300410361), and the Henan Province Medical Science and Technology Public Relations Plan Province Department joint construction project (No. SBGJ202102100).

## Acknowledgments

We thank Dr. Peng Peng for synthesizing and providing materials for this research. We acknowledge assistance with the access of analytic instruments from the Translational Medical Center at The First Affiliated Hospital of Zhengzhou University.

## Conflict of interest

The authors declare that the research was conducted in the absence of any commercial or financial relationships that could be construed as a potential conflict of interest.

## Publisher’s note

All claims expressed in this article are solely those of the authors and do not necessarily represent those of their affiliated organizations, or those of the publisher, the editors and the reviewers. Any product that may be evaluated in this article, or claim that may be made by its manufacturer, is not guaranteed or endorsed by the publisher.

## Abbreviations

COP, covalent organic polymer; PDT, photodynamic therapy; PTT, photothermal therapy; ROS, reactive oxygen species; PS, photosensitizer; DHE, dihydroethidium; NIR, near-infrared region; TEM, transmission electron microscope; HAADF-STEM, high-angle annular dark-field scanning transmission electron microscope; EDS, energy-dispersive spectrum.
